# Complete mitochondrial genome of the hydrozoan jellyfish *Blackfordia virginica* Mayer, 1910 (Cnidaria; Hydrozoa; Leptothecata) with phylogenetic analysis

**DOI:** 10.1080/23802359.2021.1903363

**Published:** 2021-03-24

**Authors:** Yoseph Seo, Jinho Chae, Jang-Seu Ki

**Affiliations:** a Department of Biotechnology, Sangmyung University, Seoul, South Korea; b Marine Environmental Research and Information Laboratory, Gunpo, South Korea

**Keywords:** Hydrozoa, *Blackfordia virginica*, mitochondrial genome, molecular phylogeny tree

## Abstract

In this study, we analyzed the complete mitochondrial genome of the hydrozoan jellyfish *Blackfordia virginica*. The genome was a linear form (15,109 bp long, 73.6% AT), including 13 protein-coding genes (*cox2, atp8, atp6, cox3, nad2, nad5, nad6, nad3, nad4L, nad1, nad4, cytB*, and *cox1*), 2 tRNAs (tRNA-Met and tRNA-Trp), and 2 rRNAs (12S and 16S RNA). The genome structure of the *B. virginica* was completely identical to mitochondrial genomes of other hydrozoans that belonged to Leptothecata and Anthoathecata. Molecular phylogenetic analysis within hydrozoan species showed that *B. virginica* was the closest to the hydrozoan *Laomedea flexuosa*.

## Introduction

Leptothecata (thecate hydroids) is an order of hydrozoans in the phylum Cnidaria. They show great morphological variations among species according to their mode of development, growth stages, and defensive structures (Maronna et al. 2016). The hydrozoans have a complex life cycle, including a polyp stage, a medusa stage, or both, and their polyps are always living in colonies which grow rapidly on rocks and shells. Based on the morphology, their taxonomy has been described for a long time (Cornelius 1995a, 1995b), and until now approximately 2300 species of Leptothecata have been documented in public database (WoRMS 2020). Recent molecular phylogenetic approaches have improved the deep relationships among species of the Leptothecata, suggesting the addition of new clades in this order (Maronna et al. 2016). Considering the huge numbers of species, there are insufficient molecular data for a more accurate classification of the true relationship.

The hydrozoan jellyfish *Blackfordia virginica* Mayer, 1910 (Cnidaria; Hydrozoa) is a member of the Leptothecata (WoRMS 2020). It was first described from the Black Sea and has been considered a native species there. However, to date, it is considered an invasive species due to the worldwide expansion via trading by ships (Mills and Sommer 1995). In this study, we first described and analyzed the complete mitochondrial genome structure of *B. virginica*. In addition, molecular phylogenetic analysis was performed using five hydrozoans, including three Leptothecata.

The specimen of *B. virginica* was collected from Songsangyo (37°12′22.4″N, 127°01′24.2″E) in South Korea, on 7 July 2020. Genomic DNA was extracted from the whole body by using the cetyl trimethylammonium bromide (CTAB) method (Richards et al. 2003) and the remaining part of the specimen was stored in the Department of Biotechnology, Sangmyung University, Korea (Accession No. EN424). The whole mitochondrial genome was sequenced on MGISEQ-200 platforms, and paired-end reads of mitogenome sequences were assembled and annotated using Getorganelle version 1.7.1a (Jin et al. 2020), MITOS (Bernt et al. 2013), and Geneious version 9.1.3 (Geneious, Auckland, New Zealand), respectively. A molecular phylogeny tree was constructed based on concatenated amino acid sequences of 13 protein-coding genes (PCGs) in MEGA X (Kumar et al., 2018). The molecular phylogenetic analysis method has been described in our previous study (Karagozlu et al. 2019).

The total length of the complete mitochondrial genome of *B. virginica* was evaluated as 15,109 bp in length (GenBank No. MW376866; 31.6% A, 42% T, 12.3% C, and 14.1% G). The genome contained 13 PCGs (*cox2, atp8, atp6, cox3, nad2, nad5, nad6, nad3, nad4L, nad1, nad4, cytB*, and *cox1*), 2 rRNAs (12S and 16S rRNA), and 2 tRNAs (tRNA-Met and tRNA-Trp). The order of 17 mitochondrial genes of *B. virginica* was completely identical to other Leptothecata, such as *Eutima* sp. (MW066348) and *Laomedea flexuosa* (JN700945), and also *Clava multicornis* (JN700935) and *Turritopsis dohrnii* (KT020766) belonging to the Anthoathecata (Kayal et al. 2012). Specifically, only 16S rRNA encoded in minority strand and short *cox1* fragment (about 100 bp) was found at the end of 16S rRNA like other Leptothecata species (Kayal et al. 2012). Mitochondrial genes of *B. virginica* have one start codon (ATG) and two stop codon (TAA/TAG). The TAA stop codon was found in all the mitochondrial PCGs, except TAG in *nad5*.

**Figure 1.**
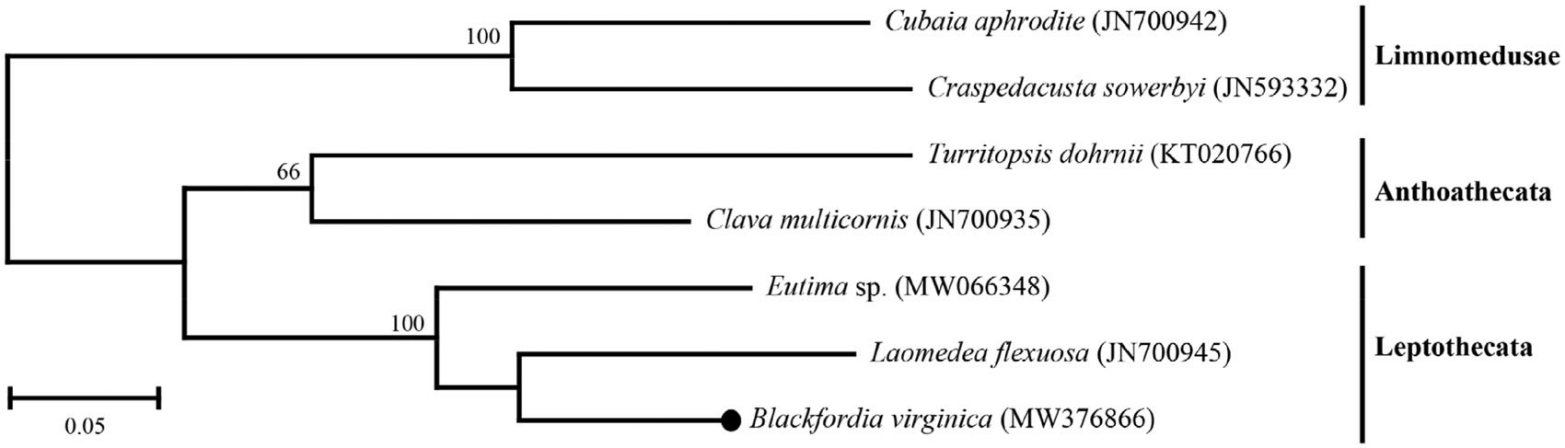
Molecular phylogenetic tree of Hydroidolina. The tree was constructed with the concatenated amino acid sequences of 13 mitochondrial protein coding genes using the maximum-likelihood algorithm (JTT matrix-based model) with 1000 bootstrap replicates. A black dot represents *Blackfordia virginica* determined in this study.

The phylogenetic relationships of the class Hydrozoa were investigated (Figure 1). The molecular phylogenetic tree showed that *B. virginica* was clustered with other Leptothecata species, and *L. flexuosa* is the closest species to *B. virginica*. The genome sequence data in this study provide additional data for phylogenetic classification among hydrozoan species.

## Data Availability

The data that support the findings of this study are openly available in GenBank with the accession number MW376866 (https://www.ncbi.nlm.nih.gov/nuccore/MW376866).

## References

[CIT0001] Bernt M, Donath A, Jühling F, Externbrink F, Florentz C, Fritzsch G, Pütz J, Middendorf M, Stadler PF. 2013. MITOS: improved de novo metazoan mitochondrial genome annotation. Mol Phylogenet Evol. 69(2):313–319. 22982435 10.1016/j.ympev.2012.08.023

[CIT0002] Cornelius PFS. 1995a. North-west European thecate hydroids and their medusae. Part 1. Introduction, Laodiceidae to Haleciidae. Synops Br Fauna. 50:1–347.

[CIT0003] Cornelius PFS. 1995b. North-west European thecate hydroids and their medusae. Part 2. Sertulariidae to Campanulariidae. Synops Br Fauna. 50:1–386.

[CIT0004] Jin JJ, Yu WB, Yang JB, Song Y, Depamphilis CW, Yi TS, Li DZ. 2020. GetOrganelle: a fast and versatile toolkit for accurate de novo assembly of organelle genomes. Genome Biol. 21(1):1–31. 10.1186/s13059-020-02154-5 PMC7488116 32912315

[CIT0005] Karagozlu MZ, Seo Y, Ki JS, Kim CB. 2019. The complete mitogenome of brownbranded moon jellyfish *Aurelia limbata* (Cnidaria, Semaeostomeae, Ulmaridae) with phylogenetic analysis. Mitochondrial DNA B. 4(1):1875–1876.

[CIT0006] Kayal E, Bentlage B, Collins AG, Kayal M, Pirro S, Lavrov DV. 2012. Evolution of linear mitochondrial genomes in medusozoan cnidarians. Genome Biol Evol. 4(1):1–12. 22113796 10.1093/gbe/evr123 PMC3267393

[CIT0007] Kumar S, Stecher G, Li M, Knyaz C, Tamura K. 2018. MEGA X: molecular evolutionary genetics analysis across computing platforms. Mol Biol Evol. 35(6):1547–1549. 29722887 10.1093/molbev/msy096 PMC5967553

[CIT0008] Maronna MM, Miranda TP, Cantero ÁLP, Barbeitos MS, Marques AC. 2016. Towards a phylogenetic classification of Leptothecata (Cnidaria, Hydrozoa). Sci Rep. 6(1):18075–18023. 26821567 10.1038/srep18075 PMC4731775

[CIT0009] Mills CE, Sommer F. 1995. Invertebrate introductions in marine habitats: two species of hydromedusae (Cnidaria) native to the Black Sea, *Maeotias inexspectata* and *Blackfordia virginica*, invade San Francisco Bay. Mar Biol. 122(2):279–288.

[CIT0010] Richards E, Reichardt M, Rogers S. 2003. Preparation of genomic DNA from plant tissue. In: Ausubel FM, Brent R, Kingston RE, Moore DD, Seidman JG, Smith JA, Struhl K, editors. Current protocols in molecular biology. New York (NY): John Wiley and Sons; p. 231–237. 10.1002/0471142727.mb0203s27 18265183

[CIT0011] WoRMS. 2020. World register of marine species. [accessed 2020 Dec 23]. http://www.marinespecies.org.

